# Perspectives of Youths on the Ethical Use of Artificial Intelligence in Health Care Research and Clinical Care

**DOI:** 10.1001/jamanetworkopen.2023.10659

**Published:** 2023-05-01

**Authors:** Kelly Thai, Kate H. Tsiandoulas, Elizabeth A. Stephenson, Dolly Menna-Dack, Randi Zlotnik Shaul, James A. Anderson, Alexis R. Shinewald, Augustina Ampofo, Melissa D. McCradden

**Affiliations:** 1 Department of Bioethics, The Hospital for Sick Children, Toronto, Ontario, Canada; 2 Genetics & Genome Biology, Peter Gilgan Centre for Research & Learning, Toronto, Ontario, Canada; 3 Labatt Family Heart Centre, The Hospital for Sick Children, Toronto, Ontario, Canada; 4 Department of Paediatrics, University of Toronto, Toronto, Ontario, Canada; 5 Holland Bloorview Kids Rehabilitation Hospital, Toronto, Ontario, Canada; 6 Institute for Health Policy, Management and Evaluation, University of Toronto, Toronto, Ontario, Canada; 7 The Hospital for Sick Children, Toronto, Ontario, Canada; 8 Dalla Lana School of Public Health, University of Toronto, Toronto, Ontario, Canada

## Abstract

**Importance:**

Understanding the views and values of patients is of substantial importance to developing the ethical parameters of artificial intelligence (AI) use in medicine. Thus far, there is limited study on the views of children and youths. Their perspectives contribute meaningfully to the integration of AI in medicine.

**Objective:**

To explore the moral attitudes and views of children and youths regarding research and clinical care involving health AI at the point of care.

**Design, Setting, and Participants:**

This qualitative study recruited participants younger than 18 years during a 1-year period (October 2021 to March 2022) at a large urban pediatric hospital. A total of 44 individuals who were receiving or had previously received care at a hospital or rehabilitation clinic contacted the research team, but 15 were found to be ineligible. Of the 29 who consented to participate, 1 was lost to follow-up, resulting in 28 participants who completed the interview.

**Exposures:**

Participants were interviewed using vignettes on 3 main themes: (1) health data research, (2) clinical AI trials, and (3) clinical use of AI.

**Main Outcomes and Measures:**

Thematic description of values surrounding health data research, interventional AI research, and clinical use of AI.

**Results:**

The 28 participants included 6 children (ages, 10-12 years) and 22 youths (ages, 13-17 years) (16 female, 10 male, and 3 trans/nonbinary/gender diverse). Mean (SD) age was 15 (2) years. Participants were highly engaged and quite knowledgeable about AI. They expressed a positive view of research intended to help others and had strong feelings about the uses of their health data for AI. Participants expressed appreciation for the vulnerability of potential participants in interventional AI trials and reinforced the importance of respect for their preferences regardless of their decisional capacity. A strong theme for the prospective use of clinical AI was the desire to maintain bedside interaction between the patient and their physician.

**Conclusions and Relevance:**

In this study, children and youths reported generally positive views of AI, expressing strong interest and advocacy for their involvement in AI research and inclusion of their voices for shared decision-making with AI in clinical care. These findings suggest the need for more engagement of children and youths in health care AI research and integration.

## Key Points

**Question:**

What are the perspectives of children and youths regarding research and clinical care involving health artificial intelligence (AI) at the point of care?

**Findings:**

In this qualitative study including 28 individuals aged 10 to 17 years, participants had a generally positive view of research, willingness to participate, knowledge about AI, engagement, and interest in AI research.

**Meaning:**

The findings of this study suggest that engaging children and youths is important to build trust and develop social license as they express a strong desire to be included in decision-making regarding AI integration.

## Teaser

This qualitive study evaluates the opinions of individuals aged 10 to 18 years on the use of artificial intelligence in research, clinical trials, and therapeutic intervention.

## Introduction

There is increasing recognition that using artificial intelligence (AI) in health care requires integration of the views and values of patients and family.^1^ Public engagement is particularly important for a technology mired in social controversy, which can undermine trust in institutions, health care professionals, and scientists.^2^ Social license supports trust by aligning values and stakeholder preferences beyond the requirements of law and regulatory bodies. For AI, social license calls on those developing and integrating AI systems to explore and understand patient values to develop value-aligned policies and practices.

Artificial intelligence in health care requires health data including routinely collected data during health encounters, such as hospital visits.^3^ The conditions in which these data are accessed and analyzed are essential to establishing and adhering to social license for AI. Adult perceptions regarding health data use for research are well characterized worldwide.^4,5,6,7,8,9,10,11,12^ Although people have a general familiarity with AI,^13,14,15,16^ AI use in the clinical context is still novel to many.^17^ Studies have reported that patients would like to take part in the development and implementation of AI and be educated about its use.^18,19^ To support AI development directed toward improving health, people are generally willing to share their health data, but privacy remains a major concern.^13,14,15,20,21^

Despite the surge of studies exploring adults’ views on the ethical issues regarding AI^1,9,13,20,22,23^ and some research in virtual care acceptance among adolescents,^24^ limited works have explored the views of children (ages, 10-12 years) and youths (ages, 13-17) regarding AI in health. Visram et al^25^ reported on a workshop they delivered to youths on a hospital advisory council that resulted in high levels of engagement, suggesting that youths are ready and willing to become involved in discussions about AI. Given that maintaining trustworthiness and social license requires understanding stakeholder views,^26,27^ it is imperative that youths are meaningfully engaged to help shape the future of health care AI. This qualitative study aimed to explore the moral perspectives and views of children and youths regarding health AI.

## Methods

This qualitative study was conducted from October 2021 to March 2022. Participants were recruited at a large urban pediatric hospital (The Hospital for Sick Children [SickKids]), purposefully sampled by selecting individuals based on demographic and health care characteristics to provide a spectrum of perspectives grounded in unique experiences. SickKids is one of the largest pediatric hospitals in the world, situated in Toronto, Ontario, where there is among the greatest population diversity in Canada. Recruitment involved posting study flyers across the hospital and outpatient clinics, social media advertising, and listing on the hospital’s research database. Interested individuals initiated first contact via email with the research coordinator (K.T.) to arrange an interview and assess eligibility. Inclusion criteria were (1) age between 10 and 17 years at recruitment when a young person is capable, (2) capable to consent (parental consent for research not required in Ontario), (3) receiving or had received care at a hospital or affiliated clinic, (4) able to communicate independently or with assistance, (5) able to access the technology to attend the interview virtually, and (6) able to communicate in English. Individuals were excluded if they were deemed not capable of consenting to participate. Verbal consent was obtained via phone by the research coordinator. Participants received compensation. Ethics approval was granted by The Hospital for Sick Children’s Research Ethics Board. This study followed the Consolidated Criteria for Reporting Qualitative Research (COREQ) reporting guideline for reporting qualitative research.

Purposive sampling was conducted to maximize diversity among participants and enabled through inclusive communication and recruitment strategies. Participants were balanced for health care experience: acute describes those who attended the hospital for urgent issues not requiring follow-up, chronic describes those with health issues requiring regular follow-up, and complex describes those with multiple health issues supported by at least 2 different care teams. In addition to health care experience, demographic characteristics (age, gender, and ethnicity) were queried. Data on ethnicity were collected to ensure our sample was representative relative to the broader Canadian population as best we could.

Our study was guided by the perspective that youths’ voices are valuable and agential, meaning that we took their moral perspectives and intuitions at face value; their experiences, voices, and values were given ethical weight.^28^ The interview was developed by the research team and designed in a vignette format, wherein participants heard fictional patients’ stories (eAppendix in Supplement 1). Interview topics were chosen to explore issues relevant to ethical use of AI, such as trust, informed consent, therapeutic misconception, therapeutic alliance, and social value. The stories were designed to touch on these topics while being relatable and realistic and featured a named protagonist to minimize social desirability bias.^29^ Interview trialing was as follows: first within the immediate research team, second with a child life specialist (A.R.S.), and third with 2 test interviews with youths who were not participants in the study.

Interviews lasted approximately 1 hour and were conducted by a single interviewer with experience in qualitative interviewing (K.T.). No repeated interviews were required. Interviews began with introductions and questions on demographic characteristics, followed by brief educational content describing AI, research, and an example that tested participants’ knowledge (eAppendix in Supplement 1). Moral intuitions were explored using 3 vignettes framed around (1) health data research (protagonist identified as River), (2) interventional AI research (protagonist identified as Latisha), and (3) clinical AI use (protagonist identified as Chad). As an exploratory study, we did not explicitly pursue thematic saturation as an end point and instead had preselected a minimum of 12 interviews across 2 groups (elementary- and high-school-age youths). We identify areas in which there were disagreements in perspectives as these will be important to guide future research.

Interviews were audiorecorded and transcribed verbatim. No repeated interviews were conducted, and transcripts were not shared with participants. Transcripts were coded and analyzed (K.T., K.H.T., and M.D.M.) thematically using Dedoose, version 9.0 software (SocioCultural Research Consultants, LLC). Transcripts were first coded by each team member independently to establish an initial set of codes; the team then met to synthesize codes and establish an initial coding framework. Two team members (K.T. and K.H.T.) led the subsequent coding and met regularly with the principal investigator (M.D.M.) during coding to ensure consistency, resolve disagreements, and discuss the developing themes. A workshop was conducted at a later date with a subset of participants to verify that the findings resonated.

## Results

In total, 44 individuals contacted the research team. Fifteen were ineligible due to age. Of the 29 who consented, 1 was lost to follow-up resulting in 28 participants (mean [SD] age, 15 [2] years, range, 10-17 years) (Table 1). The population included 6 children (age, 10-12 years) and 22 youths (age, 13-17 years). Participants identified their gender as female (16 [57%]); male (10 [36%]); and 2-spirit, trans, nonbinary, or gender diverse (3 [11%]); 7% preferred not to answer. Participants identified their ethnicity as Black (2%), East Asian (7%), mixed (7%), South Asian (18%), and White or European (54%); 7% preferred not to answer. Health care experience was relatively balanced, with 36% reporting acute health needs, 36% having chronic conditions, and 25% having complex health needs.

**Table 1.** Participant Characteristics^a^

Characteristic	No. (%)
Total	28
Age, mean (SD) [range], y	15 (2) [10-17]
Gender (self-described)	
Female or she/her	16 (57)
Male or he/him	10 (36)
Nonbinary, gender diverse, trans, or 2-spirit	3 (11)
Prefer not to answer	2 (7)
Ethnicity	
Black	2 (7)
East Asian	2 (7)
Mixed	2 (7)
South Asian	5 (18)
White/European	15 (54)
Prefer not to answer	2 (7)
Health care experience	
Acute	10 (36)
Chronic	10 (36)
Complex	7 (25)
Prefer not to answer	1 (4)

^a^ All data self-reported.

Thematic analysis was grouped as follows: AI knowledge and understanding, health data research, interventional AI research, and clinical use of AI. Some themes were revisited across stories (eg, consent). We organized our results according to the story to indicate how themes were related to the distinct stages of research. Participants were able to state their understanding of AI applications and could distinguish these from traditional computing methods. Most explained AI as a computer system that learns based on past information and the most frequently mentioned AI benefits were speed and efficiency. Participants were mostly concerned with the potential for AI systems to be hacked, have security concerns, and know too much about them. Participants reported a broad understanding of research as a process of finding information and answers. Several received formal education on AI through school or extracurricular activities. Participants broadly asserted that AI “shouldn't replace our doctors and nurses, and …can be used as a tool to benefit, but not as a replacement” (age, 16 years; female).

### River’s Story: Health Data Research

Participants endorsed an altruism-based view of health data research (Table 2). They expressed that even though this research may not benefit them individually, they would participate because of the potential to help others in the future (“I think I would [consent to participate] because if like I knew it was going to help like other people in the future, even though it, like, may not help me directly” [age, 15 years; female]). A strong theme of permission-based data access was noted: all participants but one reported that patients should be asked for permission to use their data (“It's like information about River’s self, like, so it's like River's choice … if the researchers can see it or not” (age, 12 years; male). All participants wanted information about benefits and risks and specific uses of their data.

**Table 2.** Health Data Research Themes

Theme	Quotes
Research benefits future patient	“Even if it may not benefit me directly … well, for one, it will hopefully benefit people in the future” (age, 14 y; female)
Valuing the choice to share data	“In the end it’s just … [the patient’s] decision” (age, 17 y; female)
Awareness of risks and benefits of data sharing	“I guess when [the researchers are] asking for permission, express what they specifically want to do with it and what’s their intent and how this could be helpful” (age, 17 y; female)
Relevance	“I don’t understand why a research [study] would need that [identifiable information] basically whatever is relevant to their study, I think that’s what I would provide them” (age, 13 y; male)
Confidentiality and privacy	“As long as it’s staying confidential and just within that research study…. And like, my name isn’t going to be used anywhere with this information” (age, 16 y; female)
Mental health data are more sensitive	“I’d be willing to share … physical medical conditions. I don’t know if I’d be willing to share … mental medical conditions” (age 15 y; female)
Negative views on use of social media and data from wearable devices without consent	“I feel like I would always wanna know if my information is being shared anywhere, so I’d probably be really uncomfortable” (age, 17 y; female)

Participants articulated that clinical notes and health record information were for the purpose of “understanding the patient and see what they’re going through so they can help [the patient, River] out better” (age, 12 years; male). There was a general supposition that these forms of information are kept confidential and private, which underscored their interest in researchers seeking permission to access these data. Participants expected that in research this privacy is maintained, with many asserting they would “share everything with the researchers” (age, 11 years; male). Privacy violations were linked to concerns about negative consequences; for example, one participant worried about stigma if their school had access to their health information.

Participants generally were more sensitive to research using mental health data. They reported feeling that such data are more personal and overall reported greater comfort with sharing physical health data compared with mental health data. Several grounded their hesitancy in the epistemic uncertainty involved with mental health conditions compared with physical illness: “mental health isn’t something that [is] concrete…in a sense the way a heart disease is…there isn’t no accurate…reading of it” (age, 17 years; female).

Some were comfortable with sharing all health information the researchers deemed relevant, and some only with sharing specific forms of data relevant to individual studies. There was also variation in comfort levels related to the specificity and depth of information being shared and whether they felt consent should be required for sensitive data types (eg, genetic, mental health).

Participants expressed strong negative views about social media data used for health research. Data from wearable devices and health sensors were more acceptable but only under the condition that consent was sought from the wearer. Participants reported that social media information is personal—not as in private but as in relating to the core of one’s personhood—and that they share information intentionally with family and friends (“posting stuff from your life” [age, 14 years; male]). Participants described social media information as a highly curated and biased version of themselves. Several raised the point that “it would be probably bad for researchers 'cause…there’s no confirmation that whatever you’re putting on social media is actually true” (age, 11 years; male), indicating skepticism that meaningful knowledge could be gained from using social media as a medical information source.

If researchers wanted to use data from social media, all participants but one advocated for consent to be obtained, although some remarked that this might not be feasible. One participant stated that they would be okay with researchers using their health sensor data and social media posts without them knowing “because it’s not going to harm me” (age, 17 years; male).

### Latisha: Interventional AI Research

Participants reported awareness that research typically does not benefit the participants but will benefit future patients (Table 3). They were oriented to potential risks (eg, that the AI system might not work) and, where risks were minimal, they were quite willing to participate but asserted that each patient has a right to make their own choice.

**Table 3.** Interventional AI Research Themes

Theme	Quotes
Altruism	“I would wanna help further the study and the research so that maybe another person's journey isn’t as long and painful as mine was” (age, 16 y; female)
Risks and benefits	“The first thing I want to know if there would be any drawbacks, any bad things that might happen if it doesn't work. If the whole research is, it'll help or it'll do nothing, then I would tell [patient] to go for it” (age, 14 y; female)
Vulnerability of the research participant	“If you’re really sick and somebody has a possibility of helping, like an AI … I would say yes [to research participation]” (age, 10 y; male)
Respect for persons	“[The patient’s] the one who’s like giving samples, or whatever it is that [they’re] giving to help with this research. It’s not [their] parent’s decision. [Their] parents aren’t the ones who are directly involved in this research—[the patient] is, so in the end it would be [their] decision” (age, 17 y; NR)
Sharing in decision-making	“[The patient’s] reasons are valid in terms like ‘I’m terrified this might be bad for me’” (age, 15 y; female)

Abbreviations: AI, artificial intelligence; NR, not reported.

Some participants detected a patient’s vulnerability and constrained choice, stating the patient may not be “mentally up for it” (age, 14 years; female) or able to “think rationally” (age, 13 years; male) to evaluate research participation. Most participants were aware that participation in research may or may not help a patient get better. Yet when asked what they would recommend, many still reported they would advise the patient to participate, asserting it would help the patient. Participants often associated the opportunity for research with a lack of first-line therapeutic options. Many remarked that if there were no other options, they would likely consent to research provided it presented limited harm: “It still might work, so if there's any chance at getting better then yeah, I'd probably take it because that it's gonna help me” (age, 13 years; male).

Respecting individual feelings was vital to participants regardless of capacity. They noted that decision-making should involve reflection on the patient’s thoughts and concerns, discussion with parents and the medical team, and addressing questions (“I’d ask her why she’s scared…try to get her reasoning” [age, 17 years; female]). If there was a disagreement between the prospective participant and parents, the immediate suggestion was to explore the reasoning of both perspectives. In the end, there was agreement among participants that the patient’s wishes, if reasonable, should be respected and not overridden. Participants recognized that this might be different for very young children. One participant remarked that the scientific quality could be compromised with an unwilling participant: “If she isn’t 100% willing, I would say it’s pointless…because she won’t give accurate responses and the study will not be accurate itself” (age, 17 years; female).

### Chad: Clinical Use of AI

A strong theme was the idea of being treated like a person and not a number (Table 4). Participants advocated for human interaction; for example, always seeing the patient at the bedside rather than relying exclusively on information from the AI. Some participants expressed surprise at the notion of an AI error, for example, “not because I would trust the input of an AI more, but I would just feel like it's so much more like data based, and it's so much more proficient in the sense that, like it analyzes everything it could” (age, 17 years; female). Others readily accepted the possibility of errors, stating that “the AI only knows like so much” (age, 15 years; female). Participants advocated that the physician could either check the AI after seeing the patient in person or, if they check the AI in advance, should refrain from making a decision until they actually saw the patient in person, and they overall presented a supportive view of AI (eg, “it should be more of a double checker” [age, 15 years; female]). Participants expressed 2 complementary rationales: the bedside evaluation provides essential information for making the correct decision for the patient and a personal connection is vital for respect, trust, and quality of care. For example, “the doctor needs to get like a full assessment as like a patient as a whole and like recognize that the patient is a person” (age, 16 years, female).

**Table 4.** Clinical AI Use Themes

Theme	Quotes
Being a good doctor	“If the doctor just relied on like the artificial intelligence and sort of treated Chad as like a number, you know, not like a patient, not an actual human being, then he would probably feel more frustrated ‘cause he doesn’t feel like he's being treated with like respect I guess” (age, 14 y; female)
Responsible practice	“I think like when it comes to medicine then I would be concerned that like a computer can’t know everything and it can’t take in quite as much as a human could. So whether like a doctor or nurse or another physician … the computer isn’t like present with the person the same way, so it might not acknowledge all the things that are going on with the patient as a whole” (age, 14 y; male)
Bedside presence	“I think if the doctor just goes off to the AI and then like decides off of that whatever the AI says, I think it’s really not the AI’s fault ‘cause … nothing can be perfect. But the doctor … feels kind of lazy to me for not checking anything” (age, 13 y; male)

Abbreviation: AI, artificial intelligence.

Most participants were more sympathetic toward a physician’s mistake, recognizing that physicians are human and “humans make mistakes” (age, 12 years; male). Others were more upset at AI mistakes “because if it is not accurate—it should not be used” (age, 17 years; female). Participants focused on the physician even when they attributed the mistake to the AI system because they reported that responsible use of AI was the physician’s responsibility: “I'd assume they use some of their own knowledge, but decision should not be based solely on an AI” (age, 13 years; male). Participants had a strong negative reaction if the physician did not check the patient in person but made a recommendation based on the AI alone, expressing frustration that the patient’s care was negatively impacted due to not being “properly checked up” (age, 17 years; female), calling the physician “lazy” (age, 11 years; male), and referring to “medical malpractice” (age, 13 years; male).

## Discussion

Our qualitative study describes perspectives of children and youths around ethical issues for health AI. We observed strong themes of respect for persons, support for autonomy, and humanistic care among a diverse and knowledgeable group of youths. Anecdotally, our team was impressed with the knowledge level these individuals had regarding AI and their thoughtfulness in responding. Many participants reported classes or extracurricular activities where they learned about AI. Concerns about youths’ capacity to understand the AI technology implications are, perhaps, unwarranted; given the experience of today’s youths with information technology,^29^ they may be ideally positioned to inform AI integration.

We found good overall support for both data-driven and interventional research grounded in the desire to help others, as with adults.^30^ We noted that several remarks by participants suggest therapeutic misconception—a view that the patient will derive direct clinical benefit from interventional research. Therapeutic misconception is a known problem for meaningful consent^31^ and should be addressed in the informed consent process when recruiting youths into clinical trials involving AI.

We observed a stronger emphasis on consent to data use compared with adults, where the focus instead is more on transparent communication regarding health data uses.^1,23^ The views expressed by the participants in our study are in tension with many common practices and warrant further exploration. For example, the Tri-Council Policy Statement-2 in Canada permits a research ethics board to grant a waiver of consent for research-related use of retrospective, deidentified health data. Our results suggest that even with legitimate data access, trust in AI might be compromised without addressing the gap between practices and values.

Another theme emphasized was the value of humanism in health care. The value of physician presence at the bedside is underscored by other studies.^1^ Similar to adults, children and youths are concerned about risks such as replacement of human clinicians.^1^ Many AI applications seek to streamline care, sometimes resulting in less interaction with the health care team.^32^ Going forward, balancing efficiency with relational, person-centered care will likely be crucial to nurturing a trusting relationship between clinical AI and patients. The risks we identified included increased blame attribution and a negative perception of health care professionals.

### Limitations

This study has limitations. The participants may not be representative of the general population given the recruitment from a major urban hospital and requirement of having hospital-based interactions. The interview was conducted in English, which limited participation. Acknowledging that these inclusion criteria select for a population with access to technology, research awareness, and without communication barriers to understand the study information, the team recruited from organizations promoting neuro, gender, and ethnic and racial diversity and offered all participants communication assistance tools and support persons they needed to complete the interview.

## Conclusions

In this qualitative study, we identified several opportunities for future research and education to integrate youth values in health AI, including a mismatch between data-gathering practices and values, addressing therapeutic misconception, and value misalignment regarding streamlining care delivery. We also noted several strengths, including a positive desire to be included in AI integration, strong working knowledge of AI, and a desire for a humanistic vision of medicine that includes AI. The findings of this study suggest that it may be useful for AI systems that aim to diminish contact points between health care professionals and patients be codesigned with youths and their families.

## Notes

Supplement 1. **eAppendix.** Complete Interview Guide

Supplement 2.
Data Sharing Statement
