# Supplementary material for: Perspectives of Youths on the Ethical Use of Artificial Intelligence in Health Care Research and Clinical Care
Source: JAMA Netw Open. 2023 May 1;6(5):e2310659. doi: 10.1001/jamanetworkopen.2023.10659 (PMC10152306; doi:10.1001/jamanetworkopen.2023.10659)
Supplement: Supplement 1. — eAppendix. Complete Interview Guide [file jamanetwopen-e2310659-s001.pdf]

Supplementary Online Content

Thai K, Tsiandoulas KH, Stephenson EA, et al. Perspectives of youth on the ethical use of artificial intelligence in health care research and clinical care. *JAMA Netw Open*. 2023;6(5):e2310659. doi:10.1001/jamanetworkopen.2023.10659

eAppendix. Complete Interview Guide

This supplementary material has been provided by the authors to give readers additional information about their work.

© 2023 Thai K et al. *JAMA Network Open.*

eAppendix: Complete Interview Guide

[Thank participant for participating.]

[Introduce interviewer – include pronouns, ethnicity.]

I'm going to ask you some basic demographic questions. We're asking all of our participants this because we hope to recruit a diverse sample of participants. If there is information that you don't feel comfortable sharing, you don't have to. This will not affect your participation in the research or your compensation at all.

- How old are you? (do you go to school, what grade)
- Are you comfortable sharing your gender?
- Are you comfortable sharing your ethnicity?
- Can you tell about your experience with healthcare?
- Have you been to the hospital? If yes:
- Do you mind telling me if it was an emergency visit, for surgery, or for a chronic condition?
    - If ED visit: Did you have to see a doctor for the ED visit?
- Do you have to come to the hospital or clinic often or does someone from the hospital or clinic follow-up with you often?

[Introduce the research team members with their photos]

- How did you find out about our study?
- Do you have any questions about the study so far?

<u>AI Knowledge Clarification Scripts</u>

"AI is when we use computers to learn things in the same way that humans learn them. For example, you learned how to figure out what is a truck versus a car by seeing lots of examples and maybe your parent would keep telling you which was which. AI learns like that too – we give it lots of examples that we've named for the computer. Eventually the computer learns what makes a truck a truck and not a car. Then we can give it new trucks that it's never seen before and it will still know it's a truck."

Examples:

- Siri – listens to your words and puts them together to understand you and give you information;
- YouTube/Netflix/Amazon recommender algorithm – sees examples of what you watched and gives you more examples similar to what you like;
- iPhone – can pick out a face and looks through your other pictures to find all pictures of one person based on their face, can also put together images that appear linked to make photobooks;
- Google Maps – uses information from scanners, satellites, road reports, etc to calculate the fastest traffic route for you;
- Spotify – looks at what you listened to, liked, and searched for and recommends things it predicts you will like
- SnapChat filters – analyzes your video to locate your face to put filters on in the right spot.

© 2023 Thai K et al. *JAMA Network Open.*

- Self-driving cars

“There are some things we worry about with AI. For example, it works best on examples it sees a lot. Some kinds of medical problems either aren’t very common or we don’t know a lot about them. We also worry about bias. This is where AI might not work well because of who they are. Like, for example, their skin colour, where they and their family come from, or whether their family might not have as much money as other families.”

AI Knowledge Question probes:

- Differences between computer programming and AI
- Ask participant to explain AI back to the interviewer
- Understanding of the purpose of research (should establish that research is for knowledge generation, clinical care is for patient benefit)

<u>Story 1: River’s story – Health Data Research</u>

River is 14 years old and is at the hospital because of trouble with feeling depressed, or sad. River’s family has just moved here from another country and misses their friends and family back home. River has been feeling sadder and sadder for a long time, and River’s family decided they needed some help.

Do you know about depression?

[Prompt if required] Depression is when someone feels so sad that they can’t just make themselves feel better. They need help, whether it’s therapy, medications, time with nature or their community or other things that healthcare workers and healers can do to help people feel less sad all the time.

They took River to the hospital where the doctors (called psychiatrists) who specialize in helping people with their feelings can talk to River every day and give them medicine. They take notes on what River says so they can keep track of River’s feelings and medicines.

Do you know why doctors take notes like this? [Note: goal is to get them to realize that doctors do this to make sure patients get the best care. They want everybody on the team to know important information about the patient and don’t want to forget anything. They also want to be able to keep track of how things are going, whether what they’ve tried is working, and what has or hasn’t worked before. Make them see that the goal of doctors is help patients get better (doctors vs researchers)]

Do you know who is allowed to see this information? [answer – people in ‘circle of care’ so the group of people who are helping to care for River. Researchers aren’t helping to care for patients, so they have to get permission to see this kind of information]

A team of researchers at the hospital want to understand people’s moods better to figure out if there are specific medicines that work better for specific kinds of people. To do this research, they want to look at the medical information of people with mood issues like what River has. This includes pictures of their brain, the medications that they got, lab test results, and the notes taken by the psychiatrist.

© 2023 Thai K et al. *JAMA Network Open.*

These researchers are not River's doctors. If the researchers want this information, what do you think they should do and who needs to be asked?

Prompts: who should ask and how, doctors authority and influences, talk with parents/family/friends

How old should you be before the researchers ask you for this information before asking your parents? Or do you think there isn't an age?

If the researchers asked parents for your information, do you think they should also check with you?

Do you think that River and patients like River should be asked directly to see their medical information or do they not always need to be asked? Would you want to be asked?

A person like me called a Research Assistant is someone who explains research studies to patients. A research assistant talked to River and explained that the research for this study won't actually help River, but what they learn might help patients like River in the future. If you were River would you agree to participate? Why or why not?

What kinds of information would you be willing to share (e.g. brain scans, medications, lab test results, doctor's notes)? There would be no extra tests or things you have to do, just stuff that you're already doing when the doctor is providing care for you.

If yes, would you want to know what the researchers found? How do you think researchers can do better communicating with kids and youth about the research they do? (e.g. email, video, social media, etc.)

Some kids have a hard time talking about their feelings. Do you think some information like this is different from other information like if you had a problem with maybe your heart or your stomach?

What would make you feel more or less comfortable if you were River?

Story 2: Latisha's story – Interventional AI Research

Let's say the scientists created an AI computer program. Sometimes when we make new programs – like apps – they look good and work fine, but they don't actually make things easier or better for people who use them. In healthcare, we do research to make sure that new things we do are good for patients before we use them on everybody.

Does that make sense to you? Can you think of an example like this? If not, no worries we'll talk about Latisha's story to think this through more

To think that through a bit more, we'll talk about Latisha's story.

Latisha is a 16yr old girl who is in the intensive care unit (where you go when you're really, really sick and the doctors need to watch you very closely). She has a problem with her lungs that makes it hard for her to breathe. The doctors don't know why it's happening, so they're trying different medications and other things to see if they help Latisha get better.

© 2023 Thai K et al. *JAMA Network Open.*

Doctors and researchers have different responsibilities, but both are trying to 'do good'. What do you think is the difference between the 'good' that doctors are trying to versus the 'good' that researchers are trying to do? [Clarify the goal/role of doctors and their moral obligations: The doctors and nurses are doing everything they can to improve Latisha's health.]

One doctor is doing research where she uses AI to check out the patient's genes (or DNA), scans (the pictures that doctors take of your organs), and other information about patients to try and predict whether new kinds of medicine might work.

Latisha's doctors want to use this AI to see if they can find something that might help her. But they don't know for sure yet whether the AI is helpful or not. Latisha will have to decide to participate knowing that this new AI system might help but also might not.

If you were Latisha and your doctor told you about this AI program, what would you think about first? [Explanation if needed: Some people think that if a doctor recommends a trial, it's because the doctor believes the trial is a good thing for them. But actually, when we're doing research, we don't know yet if this way is better than what we're already doing. We're doing research to really figure that out.]

Prompts: who approaches about research, understanding about research vs regular care

Probe therapeutic misconception: Research may or may not help patients get better. If you were Latisha's friend and she asked you whether she should do it, what would you tell her?

Are there people Latisha should talk to her help make a decision?

Prompts: what should she know to help her decide, who should she talk to, where do you think she'd want to get information from, who does she trust most to help her decide, role of her doctors

What if Latisha decides not to use the AI, but her parents want her to? If you were her friend what would you tell her?

<u>Story 3: Chad's story – Clinical AI Use</u>

Now we'll talk a little about what you think should be done if mistakes happen. Let's talk about Chad's case.

Chad is 12 years old and went to the hospital for food poisoning after eating something bad that made him sick. Usually, food poisoning is mild and symptoms such as vomiting will go away without treatment. However, food poisoning caused by bacteria, can be treated with antibiotics. Chad wants to be allowed to leave the hospital to go to a friend's birthday party. Chad's doctor used an AI system to help him decide what to do. The AI system's advice was that Chad wouldn't feel better with antibiotics. Chad stayed in the hospital.

How do you feel about the doctor using AI for Chad?

© 2023 Thai K et al. *JAMA Network Open.*

How would you feel if the doctor didn't actually see Chad in person. They checked the AI system and decided how Chad should be treated? Is that okay? Do you think it's important that doctors see patients in person or do they sometimes not need to?

Chad has to stay in the hospital while his family goes homes. He is really worried about missing school and activities while staying in the hospital. After a couple days, the doctor comes to Chad and says that there was a mistake. The doctor now says Chad will do much better getting antibiotics and that he can take at home. Chad goes home with his family that day.

How do you think Chad feels?

Where do you think the mistake happened? Whose fault might it be, if anyone?

How do you think Chad's parents feel?

Before you thought of the doctor using AI for Chad as […], do you still feel this way?

<u>Follow-up questions</u>

Some AI tools are ones on your phone that you can use to manage your own health. For example, we have watches that collect information about our steps, our heartrate, our exercise levels. Doctors and researchers might find this information helpful either for helping patients with their health, but also for learning about things people do outside of the hospital and how that might influence their health. What do you think about this?

Other researchers want to see your social media information for the same reasons. What do you think about this?

We've talked a lot about AI today. I'd like to hear your thoughts and opinions, what you think is good or bad, and what you want to know more about?

<u>Debrief</u>

We've talked about a few cases now with AI that are hypothetical. We want to remind you that these things aren't happening in the hospital and lots of people including this research team are working to prevent mistakes with AI.

We also want to remind you that these cases are unrelated to your care and your doctors are providing you with the best care possible. Can you tell us what you're thinking now? Is there anything that surprised you, that worried you, that you didn't know before?

What do you think all kids should know?

How do you think kids should get information about AI?

© 2023 Thai K et al. *JAMA Network Open.*

[Thank participant. Invite participant to consent to being re-contact for additional research opportunities. Invite questions.]

© 2023 Thai K et al. *JAMA Network Open.*
