# Supplementary material for: Perspectives of Youths on the Ethical Use of Artificial Intelligence in Health Care Research and Clinical Care
Source: JAMA Netw Open. 2023 May 1;6(5):e2310659. doi: 10.1001/jamanetworkopen.2023.10659 (PMC10152306; doi:10.1001/jamanetworkopen.2023.10659)
Supplement: Supplement 2. — Data Sharing Statement [file jamanetwopen-e2310659-s002.pdf]

Data Sharing Statement

Thai. Perspectives of Youths on the Ethical Use of Artificial Intelligence in Health Care Research and Clinical Care. *JAMA Netw Open*. Published May 01, 2023. doi:10.1001/jamanetworkopen.2023.10659

Data
Data available: No

Additional Information
Explanation for why data not available: As per the study protocol, only the research team will have access to participant data. Requests for data sharing can be made to the corresponding author. Provided participant consent and research ethics board approval, the study team can share de-identified interview data.
